# Progress Toward Measles Elimination — South-East Asia Region, 2003–2020

**DOI:** 10.15585/mmwr.mm7133a2

**Published:** 2022-08-19

**Authors:** Sudhir Khanal, Ahmed M. Kassem, Sunil Bahl, Liyanage Jayantha, Lucky Sangal, Mohammad Sharfuzzaman, Anindya Sekhar Bose, Sebastien Antoni, Deblina Datta, James P. Alexander

**Affiliations:** ^1^Immunizations and Vaccines Development, World Health Organization South-East Asia Regional Office, New Delhi, India; ^2^Global Immunization Division, Center for Global Health, CDC; ^3^Immunization, Vaccines and Biologicals, World Health Organization, Geneva, Switzerland.

In 2013, member states of the World Health Organization (WHO) South-East Asia Region* (SEAR) adopted the goal of measles elimination and rubella and congenital rubella syndrome control^†^ by 2020 (*1*). In 2014, to provide impetus toward achieving this goal, the Regional Director declared measles elimination and rubella control one of the Regional Flagship Priorities. In 2019, SEAR member states declared a revised goal of eliminating both measles and rubella^§^ by 2023 (*2*). The recommended strategies to achieve elimination include 1) achieving and maintaining ≥95% coverage with 2 doses of measles- and rubella-containing vaccine in every district through routine or supplementary immunization activities^¶^ (SIAs); 2) developing and sustaining a sensitive and timely case-based surveillance system that meets recommended performance indicators**; 3) developing and maintaining an accredited laboratory network; 4) achieving timely identification, investigation, and response to measles outbreaks; and 5) collaborating with other public health initiatives to achieve the preceding four strategies. This report updates a previous report and describes progress toward measles elimination in SEAR during 2003–2020 (*3*). In 2002, coverage with the first dose of a measles-containing vaccine in routine immunization (MCV1) was 70%, and only three countries in SEAR had added a second routine dose of measles-containing vaccine in routine immunization (MCV2). During 2003–2020, all countries introduced MCV2, and estimated coverage with MCV1 increased 35%, from 65% to 88%, and coverage with MCV2 increased 1,233% from 6% to 80%. Approximately 938 million persons were vaccinated in SIAs. Annual reported measles incidence declined by 92%, from 57.0 to 4.8 cases per 1 million population, and estimated deaths decreased by 97%; an estimated 9.3 million deaths were averted by measles vaccination. By 2020, five countries were verified as having achieved measles elimination. To achieve measles elimination in the region by 2023, additional efforts are urgently needed to strengthen routine immunization services and improve measles-containing vaccine (MCV) coverage, conduct periodic high-quality SIAs, and strengthen measles case-based surveillance and laboratory capacity.

## Immunization Activities

MCV1 was introduced in all 11 countries in SEAR before 2003 (Table 1). MCV2 was introduced in three countries (Indonesia, Sri Lanka, and Thailand) before 2003; the remaining eight countries introduced MCV2 during 2003–2020.

**TABLE 1:** Estimated coverage* with the first and second dose of measles-containing vaccine, vaccination schedule,^†^ number of reported measles cases,^§^ and measles incidence,^¶^^,^** by country — World Health Organization South-East Asia Region, 2003 and 2020

Country	2003						2020						% Change, 2003–2020	
	MCV schedule^†^ and vaccine type		WHO/UNICEF estimated coverage,* %		No. of reported measles cases^§^	Measles incidence^¶,^**	MCV schedule^†^ and vaccine type		WHO/UNICEF estimated coverage,* %		No. of reported measles cases^§^	Measles incidence^¶,^**	MCV1 coverage	Measles incidence**
	MCV1	MCV2	MCV1	MCV2			MCV1	MCV2	MCV1	MCV2				
Bangladesh	M, 9 mos	—^††^	76	—^††^	4,067	29.8	MR, 9 mos	MR, 15 mos	97	93	2,410	14.4	28	−52
Bhutan	M, 9 mos	—^††^	88	—^††^	0	0.0	MMR, 9 mos	MMR, 24 mos	93	92	0	0.0	6	0
Burma^§§^	M, 9 mos	—^††^	80	—^††^	830	17.7	MR, 9 mos	MR, 18 mos	91	90	444	8.3	14	−53
India	M, 9 mos	—^††^	60	—^††^	47,147	42.2	MR, 9 mos	MR, 16–24 mos	89	81	5,604	4.0	48	−91
Indonesia	M, 9 mos	M, 7 yrs^¶¶^	74	21^¶¶^	24,457	109.6	MR, 9 mos	MR, 18 mos***	76	60	524	1.9	3	−98
Maldives	M, 9 mos	—^††^	96	—^††^	75	252.3	MR, 9 mos	MMR, 18 mos	99	96	15	29.2	3	−88
Nepal	M, 9 mos	—^††^	75	—^††^	13,344	519.6	MR, 9 mos	MR, 15 mos	87	74	388	13.2	16	−97
North Korea	M, 9 mos	—^††^	95	—^††^	0	0.0	MR, 9 mos	MR, 15 mos	99	99	0	0.0	4	0
Sri Lanka	M, 9–12 mos^†††^	MR, 3 yrs	99	90	65	3.4	MMR, 1 yr	MMR, 3 yrs	96	96	2	0.1	−3	−97
Thailand	M, 9 mos	MMR, 6 yrs	96	92	4,519	69.8	MMR, 9 mos	MMR, 2.5 yrs	96	87	NR^§§§^	—^¶¶¶^	0	—^¶¶¶^
Timor-Leste	M, 9 mos	—^††^	55	—^††^	94	101.4	MR, 9 mos	MR, 18 mos	79	78	2	1.5	44	−99
**Region overall**	**NA**	**NA**	**65**	**6**	**94,598**	**57.0**	**NA**	**NA**	**88**	**80**	**9,389**	**4.8**	**35**	**−92**

**Abbreviations:** JRF = Joint Reporting Form; M = measles; MCV = measles-containing vaccine; MCV1 = first dose of MCV in routine immunization; MCV2 = second dose of MCV in routine immunization; MMR = measles-mumps-rubella; MR = measles-rubella; NA = not applicable; NR = not reported; WHO = World Health Organization.

* Data were from WHO and UNICEF estimates, 2021 revision (as of July 2022). http://immunizationdata.who.int

^†^ As reported to WHO/UNICEF on JRFs for the year.

^§^ JRF was submitted to WHO and UNICEF by member states with the official immunization data and the number of measles cases in the country for the year.

^¶^ Measles incidence is calculated based on the reported measles cases and population by member states through WHO/UNICEF JRF.

** Cases per 1 million population.

^††^ MCV2 was not introduced into routine immunization.

^§§^ *MMWR* uses the U.S. Department of State’s short-form name “Burma”; WHO uses “Myanmar.”

^¶¶^ Subnational introduction in schools of West Java at age 7 years.

*** MCV third dose administered in schools at grade 1.

^†††^ Changed in 2011 from age 9 months to 9–12 months.

^§§§^ Thailand did not report measles case data to the JRF in 2020.

^¶¶¶^ Could not be calculated.

Countries report coverage for national and subnational MCV1 and MCV2 doses delivered through the routine immunization program to WHO and UNICEF, which use data from administrative records (vaccine doses administered divided by the estimated target population) and surveys reported by member states to estimate MCV1 and MCV2 coverage (*4*). Estimated MCV1 regional coverage increased 35%, from 65% in 2003 to 88% in 2020; five countries reported ≥95% MCV1 coverage in 2020 (Table 1) (Figure). The highest regional MCV1 coverage (94%) was reached in 2019, just before the start of the COVID-19 pandemic. Estimated MCV2 coverage increased 1,233%, from 6% in 2003 to 80% in 2020, with a peak of 83% in 2019; estimated MCV2 coverage in three countries was ≥95% in 2020. During 2003–2020, measles SIAs were conducted in all countries and reached approximately 938 million persons (Supplementary Table; https://stacks.cdc.gov/view/cdc/120144).

**FIGURE:**
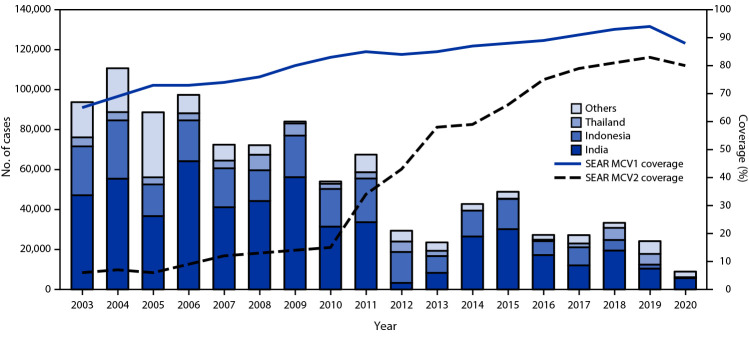
Number of reported measles cases,* by country,^†^^,^^§^ and estimated percentage of children who received their first and second dose of measles-containing vaccine^¶^ — World Health Organization South-East Asia Region, 2003–2020 **Abbreviations**: MCV = measles-containing vaccine; MCV1 = first dose of MCV in routine immunization; MCV2 = second dose of MCV in routine immunization; SEAR = South-East Asia Region; WHO = World Health Organization. * Cases of measles reported to WHO and UNICEF through the Joint Reporting Form from WHO-SEAR. ^†^ Others include Bangladesh, Bhutan, Burma, Maldives, Nepal, North Korea, Sri Lanka, and Timor-Leste. ^§^ *MMWR* uses the U.S. Department of State’s short-form name “Burma”; WHO uses “Myanmar”. ^¶^ Data were from WHO and UNICEF estimates, 2021 revision (as of July 2022). http://immunizationdata.who.int

## Surveillance Activities

By 2020, case-based measles surveillance with laboratory confirmation of suspected cases^††^ was implemented in all countries in SEAR. A measles-rubella laboratory network was established in the region by 2003 as an integral component of the WHO Global Measles and Rubella Laboratory Network. By 2020, the regional laboratory network included 49 proficient laboratories^§§^ with one regional reference laboratory (in Thailand); all countries had at least one proficient laboratory. In 2019, eight of 11 member states achieved the sensitivity indicator target of ≥2 discarded^¶¶^ measles cases per 100,000 population, and the regional discard rate was 1.68. In 2020, however, only five countries achieved the target discard rate of ≥2 per 100,000 population, and the regional discard rate was 0.98.

## Reported Measles Incidence and Measles Virus Genotypes

During 2003–2020, the number of reported*** measles cases decreased 90%, from 94,598 (2003) to 9,389 (2020). Annual measles incidence decreased 92%, from 57.0 cases per 1 million population to 4.8 cases per 1 million population (Table 1) (Figure).

Among isolates from patients during 2017–2020, measles virus genotypes detected and reported in the region included D8 in the nine countries with endemic measles^†††^; B3 in Bangladesh, Burma,^§§§^ India, Sri Lanka, and Thailand; D4 mainly in India; and H1 in Burma, India, Sri Lanka, and Thailand. However, genotype information is available for fewer than 1% of all confirmed measles cases in the region.

## Measles Case and Mortality Estimates

A previously described model for estimating measles cases and deaths (*5*,*6*) was updated with recent data for countries in SEAR. Based on the updated model, the estimated number of measles cases decreased 84%, from 16,225,870 in 2003 to 2,552,584 in 2020; estimated annual measles deaths decreased 97%, from 163,044 to 5,649 (Table 2). During 2003–2020, compared with no vaccination, measles vaccination averted an estimated 9.3 million deaths in the region.

**TABLE 2:** Estimated number of measles cases and deaths,* by country — World Health Organization South-East Asia Region, 2003–2020^†^

Country	Estimated no. of measles cases (95% CI)		Estimated no. of measles deaths (95% CI)		Estimated reduction, % 2003–2020		Cumulative no. of measles deaths averted by vaccination, 2003–2020 (95% CI)
	2003	2020	2003	2020	Measles cases	Measles deaths	
Bangladesh	874,838 (794,238–1,102,424)	322,731 (44,721–625,438)	5,969 (5,484–7,389)	454 (63–892)	63	92	712,715 (537,975–905,653)
Bhutan	1,299 (442–3,404)	524 (108–1,180)	8 (3–20)	1 (0–2)	60	88	1,635 (1,282–2,012)
Burma^§^	226,184 (195,311–263,080)	120,944 (104,245–140,792)	2,659 (2,293–3,056)	465 (402–538)	47	83	541,464 (439,755–653,704)
India	13,402,107 (11,154,888–24,654,928)	1,442,956 (1,247,122–1,623,281)	146,724 (123,133–268,096)	3,509 (3,122–3,889)	89	98	6,531,078 (5,112,728–7,919,715)
Indonesia	1,246,487 (541,014–1,930,834)	454,063 (77,520–1,209,218)	4,170 (2,549–7,759)	681 (137–1,912)	64	84	1,256,352 (1,012,703–1,515,588)
Maldives	710 (160–1,783)	112 (4–273)	NA^¶^ (0–1)	NA^¶^	84	NA	62 (46–79)
Nepal	284,033 (84,060–524,799)	182,663 (16,196–259,162)	3,075 (919–5,638)	506 (48–701)	36	84	231,909 (193,698–266,911)
North Korea	66,795 (12,907–170,701)	6,019 (2,245–14,544)	168 (33–426)	7 (3–16)	91	96	3,382 (1,756–4,555)
Sri Lanka	325 (163–1,300)	10 (5–40)	NA^¶^	NA^¶^	97	NA	44,962 (35,933–55,278)
Thailand	122,621 (102,377–136,307)	22,506 (17,145–28,182)	271 (228–305)	27 (21–34)	82	90	6,459 (4,474–8,577)
Timor-Leste	470 (235–1,880)	55 (28–220)	NA^¶^	NA^¶^	88	NA	9,228 (7,066–11,626)
**Region overall**	**16,225,870 (12,885,794–28,791,441)**	**2,552,584 (1,509,338–3,902,331)**	**163,044 (134,642–292,689)**	**5,649 (3,796–7,984)**	**84**	**97**	**9,339,246 (7,347,415–11,343,699)**

**Abbreviations**: NA = not applicable; WHO = World Health Organization.

* A measles mortality model was used to generate estimated measles cases and deaths using the WHO/UNICEF estimates of national immunization coverage data, as well as updated surveillance data. https://doi.org/10.1016/S0140-6736(12)60522-4

^†^ Data were from WHO and UNICEF estimates, 2021 revision (as of July 2022). http://immunizationdata.who.int

^§^ *MMWR* uses the U.S. Department of State’s short-form name “Burma”; WHO uses “Myanmar.”

^¶^ Estimated measles mortality was too low to allow reliable measurement of mortality reduction.

## Regional Verification of Measles Elimination

The WHO South-East Asia Regional Verification Commission for measles and rubella elimination was established in 2016 and developed a framework for verification of measles and rubella elimination in the region (*7*). Subsequently, national verification committees have been established in all 11 countries; the national committees have provided annual reports on progress toward measles elimination. As of 2020, the Regional Commission has verified measles elimination in Bhutan (2017), Maldives (2017), North Korea (2018), Sri Lanka (2019), and Timor-Leste (2018).

## Discussion

During 2003–2020, substantial progress was made toward measles elimination in SEAR. Through implementation of the regional strategies, estimated MCV1 and MCV2 coverage increased 35% and 1,233%, respectively; reported measles incidence declined by 92%; and estimated measles deaths decreased by 97%. By the end of 2019, five of the 11 countries had been verified as having eliminated endemic measles transmission.

In September 2019, after an extensive review of the progress made and the biologic, programmatic, and financial feasibility of measles and rubella elimination, the member states in the region updated the goal to achieve measles and rubella elimination by 2023 (*2*). However, challenges to achieving measles elimination in SEAR exist. During the COVID-19 pandemic, routine MCV1 coverage in the region declined from a peak of 94% in 2019 to 88% in 2020, and MCV2 coverage declined from a peak of 83% (2019) to 80% (2020). In 2020, among the estimated 22.3 million infants who did not receive MCV1 worldwide, approximately 18% were from SEAR, including 3 million in India and 0.6 million in Indonesia (*4*). In addition, measles surveillance sensitivity declined in all countries in the region, perhaps because COVID-19 mitigation measures (e.g., physical distancing and masking) decreased transmission of measles and other respiratory viruses but also because of reductions in clinic visits for febrile rash illness resulting from movement restrictions imposed nationally and the deployment of surveillance staff members to respond to the COVID-19 pandemic. A recent independent review of progress toward measles elimination in SEAR (*8*) concluded that several challenges, including immunity gaps, suboptimal sensitivity of surveillance, inadequate outbreak response and preparedness, funding gaps, and the negative effects of the COVID-19 pandemic on immunization programs threaten achievement of the 2023 target.

The findings in this report are subject to at least four limitations. First, coverage estimates are based on administrative data and might be inaccurate because of errors in recording of doses administered or in estimates of the target populations. Second, surveillance data might underestimate true disease incidence because not all patients seek care and not all measles cases in patients who seek care are reported. Third, genotype data are based on a limited number of sequences and might not reflect the predominant genotypes in the region. Finally, the measles estimation model might be inaccurate because of errors in the immunization coverage estimates and reported cases as well as the inherent uncertainty of estimates based on modeling.

Achieving measles elimination in SEAR by 2023 will require urgent intensified efforts by countries to implement strategies optimally and in a very short period, especially to mitigate the deleterious effects of the COVID-19 pandemic on immunization services. The 2023 target date represents an opportunity to re-energize efforts and maintain momentum in the region to 1) obtain the highest level of political commitment from member states and support from partners; 2) strengthen routine immunization and achieve ≥95% coverage with MCV1 and MCV2; 3) conduct high-quality SIAs; 4) enhance surveillance sensitivity and increase collection of specimens for measles virus detection and genotyping; and 5) leverage measles elimination activities to enhance efforts to restore immunization services and reduce gaps in immunity to all vaccine-preventable diseases in recovery from the COVID-19 pandemic. As of 2020, all 11 countries in SEAR had developed national plans for elimination based on strategies outlined in the Global Measles and Rubella Strategic Plan (*9*) and the regional committee resolution (*2*). With 34.3 million surviving infants in SEAR (24% of the global total), regional measles elimination represents a substantial opportunity to decrease measles-related death and illness worldwide by 2023 (*1*,*6*,*8*).

**Summary**

**What is already known about this topic?**

In 2002, coverage with the first dose of measles-containing vaccine (MCV1) in the World Health Organization’s South-East Asia Region (SEAR) was 70%, but only three countries had added a second routine dose of measles-containing vaccine (MCV2).

**What is added by this report?**

During 2003–2020, all countries in SEAR introduced MCV2, and estimated MCV1 and MCV2 coverage increased from 65% to 88% and from 6% to 80%, respectively. Reported measles incidence declined by 92%; measles vaccination averted an estimated 9.3 million deaths. Five countries achieved measles elimination by 2020, and the region adopted a 2023 goal of measles and rubella elimination.

**What are the implications for public health practice?**

To achieve measles elimination in SEAR by 2023, additional efforts are urgently needed to strengthen routine immunization services and improve measles-containing vaccine coverage, conduct periodic high-quality supplementary immunization activities, and strengthen measles case-based surveillance and laboratory capacity.

## Footnotes

* The WHO SEAR consists of 11 countries: Bangladesh, Bhutan, Burma, India, Indonesia, Maldives, Nepal, North Korea, Sri Lanka, Thailand, and Timor-Leste.

† Measles elimination is defined as the absence of endemic measles cases for a period of ≥12 months in the presence of adequate surveillance. Rubella and congenital rubella syndrome control is defined as 95% reduction in disease incidence from the 2013 level.

§ Rubella elimination is defined as the absence of endemic rubella cases for a period of ≥12 months in the presence of adequate surveillance.

¶ SIAs are generally conducted using two target age ranges. An initial, nationwide catch-up SIA focuses on all children and adolescents aged 9 months–14 years, with the goal of eliminating susceptibility to measles in the general population. Follow-up SIAs are generally conducted nationwide every 2–4 years and target children aged 9–59 months with the goal of eliminating any measles susceptibility that has developed in recent birth cohorts and protecting children who did not respond to the first measles-containing vaccine dose.

** These indicators include 1) ≥2 discarded nonmeasles nonrubella cases per 100,000 population at the national level per year; 2) ≥2 discarded nonmeasles nonrubella cases per 100,000 population per year in ≥80% of subnational administrative units; 3) testing of ≥80% of suspected measles cases for measles immunoglobulin M antibodies; 4) adequate investigation conducted within 48 hours of notification of ≥80% of suspected cases; 5) adequate collection of samples for detecting measles or rubella viruses and testing in accredited laboratory of ≥80% of laboratory-confirmed chains of transmission; and 6) an annualized incidence rate of zero for confirmed endemic measles cases.

†† The definition of a suspected measles case was “acute fever with maculopapular rash” in nine member states and “fever and rash with cough, coryza or conjunctivitis” in the other two.

§§ A laboratory that has met defined criteria as outlined in the report, “Framework for verifying elimination of measles and rubella.” https://www.who.int/wer

¶¶ A discarded case is defined as a suspected case that has been investigated and determined to be neither measles nor rubella using 1) laboratory testing in a proficient laboratory or 2) epidemiologic linkage to a laboratory-confirmed outbreak of another communicable disease that is not measles or rubella. The discarded case rate is used to measure the sensitivity of measles surveillance.

*** Countries report the number of incident measles cases to WHO and UNICEF annually using the Joint Reporting Form.

††† Bhutan and Maldives eliminated endemic measles transmission in 2017.

§§§ *MMWR* uses the U.S. Department of State short-form name “Burma”; WHO uses “Myanmar.”
